# Tobacco Product Use Among Adults — United States, 2012–2013

**Published:** 2014-06-27

**Authors:** Israel T. Agaku, Brian A. King, Corinne G. Husten, Rebecca Bunnell, Bridget K. Ambrose, S. Sean Hu, Enver Holder-Hayes, Hannah R. Day

**Affiliations:** 1 Office on Smoking and Health, National Center for Chronic Disease Prevention and Health Promotion, CDC; 2 EIS officer, CDC; 3 Center for Tobacco Products, Food and Drug Administration

Despite significant declines in cigarette smoking among U.S. adults over the past five decades, progress has slowed in recent years, and the prevalence of use of other tobacco products such as cigars and smokeless tobacco has not changed (1,2). Additionally, the prevalence of use of emerging products, including electronic cigarettes (e-cigarettes), has rapidly increased (3). This report provides the most recent national estimates of tobacco use among adults aged ≥18 years, using data from the 2012–2013 National Adult Tobacco Survey (NATS). The findings indicate that 21.3% of U.S. adults used a tobacco product every day or some days, and 25.2% used a tobacco product every day, some days, or rarely. Population-level interventions focused on the diversity of tobacco product use, including tobacco price increases, high-impact antitobacco mass media campaigns, comprehensive smoke-free laws, and enhanced access to help quitting, in conjunction with Food and Drug Administration (FDA) regulation of tobacco products, are critical to reducing tobacco-related diseases and deaths in the United States (4).

The 2012–2013 NATS is a stratified, national random-digit–dialed landline and cellular telephone survey of 60,192 noninstitutionalized U.S. adults aged ≥18 years. The response rate to the survey was 44.9% (landline = 47.2%, cellular = 36.3%). The survey assessed use of the following tobacco product types: cigarettes, cigars/cigarillos/filtered little cigars, regular pipes, water pipes/hookah, e-cigarettes; chewing tobacco/snuff/dip, snus, and dissolvable tobacco products. Based on documented differences in the patterns of tobacco product use (1), NATS assessed varying thresholds of lifetime use to separate established users from experimenters and nonusers. Usage thresholds for the different tobacco product types were as follows: cigarettes (≥100 times), cigars/cigarillos/filtered little cigars (≥50 times), regular pipes (≥50 times), water pipes/hookahs (≥1 time), chewing tobacco/snuff/dip (≥20 times), e-cigarettes (≥1 time), snus (≥1 time), and dissolvable tobacco products (≥1 time). Respondents who met the respective thresholds were then asked if they now used the product “every day,” “some days,” or “not at all.” A response option of “rarely” was also provided for all tobacco products other than cigarettes based on cognitive testing suggesting that some users of these other products did not consider “some days” or “not at all” to accurately reflect their use pattern. Because of limited sample size, all smokeless tobacco products (chewing tobacco/snuff/dip, snus, and dissolvable tobacco products) were aggregated into a single category.

Data were weighted to provide nationally representative estimates. Two definitions were used to assess the effect of occasional tobacco use on estimates of current tobacco use: 1) every day or some days, and 2) every day, some days, or rarely. Any tobacco product use was defined as use of at least one tobacco product type.* Any combustible tobacco product use was defined as use of at least one of the following tobacco product types: cigarettes, cigars/cigarillos/filtered little cigars, regular pipes, or water pipes/hookah. Tobacco use prevalence estimates were calculated overall and by sex, age, race/ethnicity, U.S. Census region,† education, annual household income, and sexual orientation. Prevalence estimates with a relative standard error ≥30% were omitted. Differences between groups were assessed using chi-squared statistics (p<0.05).

The percentages of all respondents who had ever met the threshold for each product type (i.e., current and former users), were as follows: cigarettes, 43.1%; cigars/cigarillos/filtered little cigars, 12.6%; regular pipes, 5.0%; water pipes/hookahs, 12.3%; e-cigarettes, 14.1%; chewing tobacco/snuff/dip, 9.6%; dissolvable tobacco products, 0.4%; and snus, 5.4%.

During 2012–2013, an estimated 21.3% of U.S. adults used any tobacco product every day or some days (73.4% of these used ≥1 tobacco products daily), and 19.2% used any combustible tobacco product every day or some days (72.1% of these used ≥1 combustible tobacco products daily) (Table 1). Prevalence of every day or some days use of specific tobacco products was as follows: cigarettes, 18.0%; cigars/cigarillos/filtered little cigars, 2.0%; regular pipes, 0.3%; water pipes/hookah, 0.5%; e-cigarettes, 1.9%; smokeless tobacco, 2.6%. An estimated 25.2% of U.S. adults reported now using any tobacco product every day, some days, or rarely (62.7% of these used ≥1 tobacco products daily), and 22.9% used any combustible tobacco product every day, some days, or rarely (60.6% of these used ≥1 combustible tobacco products daily) (Table 2). Prevalence of every day, some days, or rarely use was as follows: cigars/cigarillos/filtered little cigars, 5.8%; regular pipes, 0.9%; water pipes/hookah, 3.9%; e-cigarettes, 4.2%; smokeless tobacco, 3.8%. Prevalence of every day, some days, or rarely use was significantly higher than every day or some day use for any tobacco product use, cigars/cigarillos/filtered little cigars, regular pipes, water pipes/hookah, e-cigarettes, and smokeless tobacco (p<0.05).

Among respondents who had ever met the threshold for each product type (i.e., current and former users), current everyday use was as follows: cigarettes, 30.9%; cigars/cigarillos/filtered little cigars, 5.8%; regular pipes, 2.2%; water pipes/hookahs, 0.4%; e-cigarettes, 5.3%; chewing tobacco/snuff/dip, 17.1%; dissolvable tobacco products, 3.1%; and snus, 1.8% (Figure). Among respondents who had ever met the threshold for each product type and who now used the product (i.e., current users only), current everyday use was as follows: cigarettes, 74.2%; cigars/cigarillos/filtered little cigars, 12.8%; regular pipes, 12.6%; water pipes/hookahs, 1.2%; e-cigarettes, 17.9%; chewing tobacco/snuff/dip, 48.1%; dissolvable tobacco products, 16.8%; and snus, 11.3%.

By sex, prevalence of any tobacco use every day or some days was higher among men (26.2%) than women (15.4%) (Table 1). By age, prevalence was highest among those aged 25–44 years (25.2%) and lowest among those aged ≥65 years (9.5%). By race/ethnicity, prevalence was highest among adults categorized as “other, non-Hispanic” (33.0%) and lowest among non-Hispanic Asians (8.8%). By region, prevalence was highest in the Midwest (23.9%) and lowest in the West (19.0%). Prevalence by education was highest among adults with a General Education Development certificate (43.8%) and lowest among those with a graduate degree (6.3%). Prevalence was highest among adults with annual household income of <$20,000 (29.8%) and lowest among those with income ≥$100,000 (12.8%). By sexual orientation, prevalence was higher among lesbian, gay, bisexual, or transgender (LGBT) adults (30.8%) than heterosexual/straight adults (20.5%).

## Discussion

During 2012–2013, an estimated one in five U.S. adults (50 million persons) currently used any tobacco product every day or some days, and an estimated one in four (60 million persons) used tobacco products every day, some days, or rarely. Any tobacco use was greater among men, younger adults, non-Hispanic other adults, those living in the Midwest and South, those with less education and income, and LGBT adults. Continued implementation of proven population-based interventions, including increasing tobacco product prices, implementing and enforcing comprehensive smoke-free laws, warning about the dangers of tobacco use through high-impact mass media campaigns, and increasing access to help quitting, can help reduce tobacco use (1,4,5). Additionally, regulatory authority over the manufacture, marketing, and sales of tobacco products are powerful tools to further reduce tobacco-related disease and deaths in the United States.§ In April 2014, FDA proposed to extend its authority to additional tobacco products, including e-cigarettes, cigars, pipes, and water pipes/hookahs.¶ This proposed rule would set a national minimum age for sales; require health warnings, tobacco ingredient reporting, and listing of harmful and potentially harmful constituents; ensure FDA premarket review of new and changed tobacco products and all marketing of reduced risk products; and enable future rulemaking regarding product manufacture, marketing, and sales.

Although the prevalence of every day or some day cigarette smoking (18.0%) was significantly lower than the prevalence observed in the 2009–2010 NATS (19.5%) (6), cigarettes and other combustible products (e.g., cigars, pipes, and hookahs) remained the most prevalent forms of adult tobacco use. The 50th anniversary Surgeon General’s report on the health consequences of smoking concluded that disease and deaths from tobacco use are overwhelmingly caused by cigarettes and other combusted products, and that rapid elimination of their use will dramatically reduce this burden (1). Additionally, the use of emerging tobacco products (e.g., e-cigarettes and water pipes/hookahs) was also evident and could be attributed to lower price relative to cigarettes; an increase in marketing, availability, and visibility; and the perception that they might be safer alternatives to cigarettes (1). Taken together, these findings underscore the importance of continued implementation of proven population-based interventions to address all forms of tobacco use, especially combustible products that currently account for the greatest public health burden.

Accounting for respondents who reported rarely using each respective tobacco product resulted in higher prevalence estimates among all population subgroups, especially young adults. A sensitivity analysis using NATS data showed that young adults were more likely to report using any tobacco products rarely. However, it cannot be determined from these data whether this represents early initiation that will escalate to established use. Furthermore, omitting the lifetime thresholds used to identify established users yielded higher estimates for certain products, including cigars/cigarillos/filtered little cigars. For example, overall use of cigars/cigarillos/filtered little cigars every day, some days, or rarely was 5.8% using the 50 lifetime cigar threshold and 7.4% without. Hence, intensified efforts are warranted to monitor occasional tobacco use in population-level surveys and to enhance the accuracy and sensitivity of tobacco use measures, particularly among young adults.

The findings in this report are subject to at least four limitations. First, self-reported tobacco use might have resulted in misreporting; however, self-reported cigarette smoking correlates highly with serum cotinine levels (7). Second, small sample sizes for certain subgroups resulted in less precise estimates. Third, the response rate of 44.9% might have resulted in nonresponse bias, even after adjustment for nonresponse. Fourth, the established thresholds and current use measures varied by tobacco product type. Although not a limitation, it is important to note that these estimates might differ from those derived from other surveillance systems. For example, although estimates of cigarette smoking from NATS were comparable with the National Health Interview Survey (NHIS) (8), the National Survey on Drug Use and Health (NSDUH) consistently yields higher estimates than NATS and NHIS (9). These differences might be explained, in part, by varying survey methodologies and tobacco use definitions. For example, NSDUH is conducted completely in-person, uses a self-administered survey mode, and provides incentives to participants (10).

Sustained, comprehensive state tobacco control programs funded at CDC-recommended levels can accelerate progress toward reducing tobacco-related diseases and deaths in the United States (4). However, during 2014, despite combined revenue of more than $25 billion from settlement payments and tobacco taxes for all states, states will spend only $481.2 million (1.9%) on comprehensive tobacco control programs,** representing <15% of the CDC-recommended level of funding for all states combined (4). Full implementation of comprehensive tobacco control programs at CDC-recommended funding levels, in conjunction with FDA regulation of tobacco products, could reduce tobacco use and change social norms regarding the acceptability of tobacco use in the United States (1,4,5).

What is already known on this topic?

Despite declines in cigarette smoking among U.S. adults, the use of other tobacco products (e.g., cigars and smokeless tobacco) has not changed. Additionally, the use of emerging products, including electronic cigarettes, has rapidly increased.

What is added by this report?

During 2012–2013, an estimated 21.3% of U.S. adults (50 million persons) reported use of any tobacco product every day or some days, and 25.2% (60 million persons) reported use every day, some days, or rarely. Variations in any tobacco use were observed across population groups; prevalence was greater among men, younger adults, non-Hispanic other adults, those living in the Midwest and South, those with less education and income, and lesbian, gay, bisexual, or transgender adults.

What are the implications for public health practice?

The findings in this report underscore the importance of continued implementation of proven population-based interventions focused on the diversity of tobacco product use in the United States. Such interventions include increasing tobacco product prices, implementing and enforcing comprehensive smoke-free laws, warning about the dangers of tobacco use through high-impact antitobacco mass media campaigns, and increasing access to help quitting, in conjunction with Food and Drug Administration regulation of tobacco products. Sustained, comprehensive state tobacco control programs funded at CDC-recommended levels can accelerate progress toward reducing tobacco-related diseases and deaths in the United States.

## Figures and Tables

**FIGURE:**
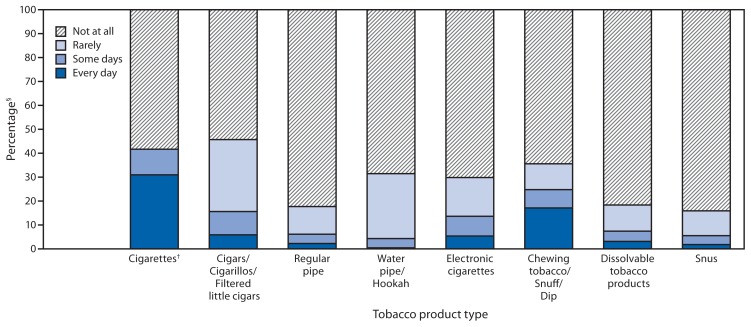
Percentage of persons who used selected tobacco products among those who met established thresholds,* by product type and frequency of use — National Adult Tobacco Survey, United States, 2012–2013 **Note:** Denominator for each product included respondents who had ever reached the threshold for the specified product (including current and former users). * Thresholds for the respective products were determined by asking the respondents if they had used the product a specified number of times. Frequency of cigarette smoking was determined among respondents who reported smoking ≥100 cigarettes during their lifetime (n = 26,381); frequency of cigar/cigarillos/ filtered little cigar smoking was determined among respondents who reported smoking the product ≥50 times during their lifetime (n = 6,687); frequency of regular pipe smoking was determined among respondents who reported smoking the product ≥50 times during their lifetime (n = 3,813); frequency of chewing tobacco, snuff, or dip use was determined among respondents who reported using the products ≥20 times during their lifetime (n = 5,004); frequency of water pipe/hookah (n = 4,924), electronic cigarettes (n = 5,905), snus (n = 2,337), and dissolvable tobacco products (n = 152) was determined among respondents who reported using these products at least one time during their lifetime. ^†^ Cigarettes were the only tobacco product type for which frequency of use was assessed with the response options “every day,” “some days,” or “not at all.” All other tobacco product types were assessed with four response options: “every day,” “some days,” “rarely,” or “not at all.” ^§^ The frequency distribution of cigarette usage at the time of the survey among those who had ever met the threshold was as follows: everyday (30.9%), some days (10.8%), or not at all (58.3%). For all other tobacco products, frequency distribution of usage at the time of the survey for everyday, some days, rarely, or not at all, respectively, among those who had ever met the respective thresholds was as follows: cigars/cigarillos/filtered little cigars (5.8%, 9.8%, 30.1%, and 54.3%), regular pipes (2.2%, 3.9%, 11.6%, and 82.3%), water pipes/hookahs (0.4%, 3.9%, 27.1%, and 68.6%), electronic cigarettes (5.3%, 8.3%, 16.2%, and 70.2%), chewing tobacco/snuff/dip (17.1%, 7.7%, 10.8%, and 64.5%), dissolvable tobacco products (3.1%, 4.3%, 10.9%, and 81.7%), and snus (1.8%, 3.7%, 10.4%, and 84.1%).

**TABLE 1:** Percentage of persons aged ≥18 years who were “every day” or “some day” tobacco users among those who met established thresholds, by tobacco product and selected characteristics — National Adult Tobacco Survey, United States, 2012–2013

	Any tobacco product*		Any combustible tobacco product†		Cigarettes§		Cigars/Cigarillos/Filtered little cigars**		Regular pipe††		Waterpipe/hookah§§		Electronic cigarettes		Smokeless tobacco***	
								
	%	(95% CI)	%	(95% CI)	%	(95% CI)	%	(95% CI)	%	(95% CI)	%	(95% CI)	%	(95% CI)	%	(95% CI)
**Overall**	**21.3**	**(20.8–21.8)**	**19.2**	**(18.7–19.7)**	**18.0**	**(17.5–18.5)**	**2.0**	**(1.8–2.2)**	**0.3**	**(0.2–0.4)**	**0.5**	**(0.4–0.6)**	**1.9**	**(1.8–2.1)**	**2.6**	**(2.4–2.7)**
**Sex**																
Men	26.2	(25.4–27.0)	22.6	(21.8–23.4)	20.0	(19.8–21.4)	3.2	(2.8–3.5)	0.5	(0.4–0.6)	0.6	(0.5–0.8)	2.2	(1.9–2.5)	4.8	(4.4–5.2)
Women	15.4	(14.8–16.0)	14.9	(14.3–15.5)	14.5	(13.9–15.1)	0.7	(0.6–0.9)	—¶		0.4	(0.2–0.5)	1.6	(1.4–1.8)	0.3	(0.2–0.3)
**Age group (yrs)**																
18–24	24.0	(22.3–25.7)	21.3	(19.7–23.0)	18.5	(16.9–20.0)	3.4	(2.6–4.2)	0.5	(0.3–0.7)	2.5	(1.9–3.2)	2.4	(1.8–3.0)	4.4	(3.7–5.1)
25–44	25.2	(24.2–26.2)	23.0	(22.0–23.9)	21.8	(20.9–22.8)	2.3	(1.9–2.6)	0.3	(0.2–0.4)	0.5	(0.3–0.6)	2.4	(2.0–2.7)	3.1	(2.7–3.5)
45–64	22.3	(21.5–23.0)	20.2	(19.4–20.9)	19.2	(18.5–19.9)	1.7	(1.5–1.9)	0.2	(0.2–0.3)	—¶		2.0	(1.7–2.2)	2.1	(1.9–2.4)
≥65	9.5	(9.0–10.1)	8.6	(8.0–9.1)	7.8	(7.3–8.3)	0.9	(0.7–1.1)	0.3	(0.2–0.4)	—¶		0.6	(0.5–0.8)	1.0	(0.8–1.2)
**Race/Ethnicity**																
White, Non-Hispanic	20.7	(20.1–21.3)	18.2	(17.7–18.8)	17.2	(16.6–17.7)	1.6	(1.4–1.8)	0.3	(0.2–0.4)	0.4	(0.3–0.6)	2.1	(1.9–2.3)	3.0	(2.8–3.3)
Black, Non-Hispanic	22.5	(20.7–24.3)	21.6	(19.9–23.4)	19.7	(18.0–21.4)	3.7	(2.8–4.6)	—¶		—¶		0.8	(0.5–1.2)	1.0	(0.6–1.3)
Asian, Non-Hispanic	8.8	(6.2–11.3)	8.6	(6.1–11.2)	7.6	(5.3–10.0)	—¶		—¶		—¶		—¶		—¶	
Other, Non-Hispanic	33.0	(30.8–35.2)	29.8	(27.6–32.0)	27.9	(25.7–30.0)	3.7	(2.8–4.6)	0.8	(0.4–1.2)	0.9	(0.5–1.3)	3.8	(2.9–4.8)	4.4	(3.4–5.3)
Hispanic	15.9	(14.6–17.3)	15.4	(14.1–16.8)	14.6	(13.2–15.9)	1.4	(0.9–1.9)	—¶		0.6	(0.3–0.9)	1.1	(0.8–1.4)	0.6	(0.4–0.9)
**U.S. Census region** †††																
Northeast	19.7	(18.4–21.0)	18.0	(16.7–19.2)	16.0	(15.5–17.9)	1.8	(1.3–2.3)	0.5	(0.2–0.7)	0.7	(0.4–1.0)	1.8	(1.3–2.2)	1.9	(1.5–2.3)
Midwest	23.9	(22.7–25.0)	20.9	(19.8–22.0)	19.4	(18.3–20.5)	2.4	(1.9–2.8)	0.4	(0.2–0.5)	0.5	(0.3–0.7)	2.2	(1.8–2.7)	3.9	(3.3–4.5)
South	22.9	(22.0–23.8)	20.7	(19.8–21.6)	19.5	(18.6–20.3)	2.3	(2.0–2.7)	0.3	(0.2–0.3)	0.5	(0.3–0.6)	2.0	(1.7–2.3)	2.8	(2.5–3.2)
West	19.0	(18.2–19.9)	17.5	(16.7–18.3)	16.4	(15.6–17.2)	1.5	(1.3–1.8)	0.2	(0.2–0.3)	0.6	(0.4–0.7)	1.8	(1.5–2.0)	1.9	(1.7–2.2)
**Education**																
0–12 years (no diploma)	28.2	(26.3–30.1)	26.5	(24.6–28.3)	25.6	(23.7–27.4)	2.5	(1.8–3.2)	0.2	(0.1–0.3)	—¶		1.6	(1.1–2.0)	2.7	(2.1–3.3)
GED	43.8	(39.5–48.1)	42.0	(37.7–46.3)	41.0	(36.7–45.3)	3.8	(1.9–5.6)	—¶		—¶		3.1	(1.5–4.6)	3.3	(1.8–4.7)
High school diploma	24.2	(23.1–25.3)	21.4	(20.4–22.5)	20.2	(19.2–21.2)	2.2	(1.8–2.6)	0.3	(0.2–0.4)	0.7	(0.4–0.9)	2.4	(2.0–2.8)	3.5	(3.1–4.0)
Some college, no diploma	23.6	(22.4–24.9)	21.5	(20.4–22.7)	20.0	(18.8–21.1)	2.2	(1.8–2.7)	0.5	(0.2–0.7)	1.0	(0.6–1.3)	2.5	(2.0–2.9)	2.3	(1.9–2.8)
Associate degree	21.4	(20.2–22.6)	19.2	(18.0–20.4)	18.0	(16.8–19.1)	1.8	(1.4–2.3)	0.3	(0.1–0.5)	—¶		2.5	(2.0–2.9)	2.5	(2.0–2.9)
Undergraduate degree	10.9	(10.2–11.6)	9.2	(8.6–9.9)	8.2	(7.6–8.8)	1.1	(0.9–1.3)	0.2	(0.1–0.4)	0.3	(0.1–0.4)	1.0	(0.8–1.2)	1.8	(1.4–2.1)
Graduate degree	6.3	(5.7–6.9)	5.7	(5.1–6.2)	4.9	(4.3–5.4)	0.7	(0.5–0.9)	0.1	(0.1–0.2)	—¶		0.5	(0.3–0.6)	0.7	(0.4–0.9)
**Annual household income ($)**																
<20,000	29.8	(28.0–31.5)	27.9	(26.1–29.6)	26.4	(24.7–28.1)	3.8	(3.0–4.5)	0.4	(0.2–0.6)	0.6	(0.3–0.9)	2.5	(1.9–3.1)	2.2	(1.7–2.7)
20,000–49,999	25.6	(24.5–26.6)	23.7	(22.7–24.8)	22.6	(21.6–23.6)	1.9	(1.6–2.3)	0.2	(0.1–0.3)	0.5	(0.4–0.7)	1.9	(1.6–2.2)	2.5	(2.2–2.9)
50,000–99,999	19.3	(18.3–20.2)	16.9	(16.0–17.8)	15.7	(14.8–16.6)	1.6	(1.3–2.0)	0.3	(0.1–0.4)	0.5	(0.3–0.7)	2.3	(1.9–2.7)	2.8	(2.4–3.2)
≥100,000	12.8	(11.9–13.7)	10.6	(9.8–11.5)	9.3	(8.5–10.1)	1.7	(1.3–2.1)	0.2	(0.1–0.3)	0.4	(0.2–0.6)	1.4	(1.0–1.7)	2.8	(2.3–3.3)
Unspecified	20.9	(19.7–22.0)	19.0	(18.1–20.3)	18.0	(16.9–19.1)	1.8	(1.4–2.2)	0.4	(0.3–0.6)	0.6	(0.4–0.9)	1.6	(1.2–1.9)	2.2	(1.8–2.6)
**Sexual orientation**																
Heterosexual/straight	20.5	(20.0–21.1)	18.5	(18.0–19.0)	17.3	(16.8–17.8)	1.9	(1.7–2.1)	0.2	(0.2–0.3)	0.4	(0.3–0.5)	1.9	(1.7–2.1)	2.6	(2.4–2.8)
LGBT	30.8	(27.7–34.0)	29.4	(26.3–32.5)	27.7	(24.7–30.7)	3.0	(1.6–4.3)	—¶		—¶		4.5	(3.0–5.9)	1.9	(0.9–2.9)
Unspecified	24.0	(22.3–25.6)	21.9	(20.3–23.5)	20.4	(18.9–22.0)	2.6	(2.0–3.2)	0.7	(0.5–1.0)	1.1	(0.6–1.5)	1.5	(1.1–2.0)	2.7	(2.1–3.3)

**Abbreviations:** CI = confidence interval; GED = General Education Development certificate; LGBT = lesbian, gay, bisexual, or transgender.

* Any tobacco use was defined as “every day” or “some days” use of cigarettes; cigars, cigarillos, or filtered little cigars; pipes; water pipes/hookah; electronic cigarettes; or smokeless tobacco (snus, dissolvable tobacco products, snuff, chewing tobacco, or dip).

† Any combustible tobacco use was defined as “every day” or “some days” use of cigarettes; cigars, cigarillos, or filtered little cigars; pipes; or water pipes/hookah.

§ Reported smoking at least 100 cigarettes during their lifetime and now smoked “every day” or “some days.”

¶ Estimate not presented because relative standard error ≥30%.

** Reported smoking at least 50 cigars, cigarillos, or filtered little cigars during their lifetime and now smoked “every day” or “some days.”

†† Reported smoking a regular pipe filled with tobacco at least 50 times during their lifetime and now smoked “every day” or “some days.”

§§ Reported smoking tobacco in a hookah at least once during their lifetime and now smoked “every day” or “some days.”

¶¶ Reported smoking electronic cigarettes at least once during their lifetime and now smoked “every day” or “some days.”

*** Smokeless tobacco users were defined using three product types: 1) chewing tobacco, snuff, or dip; 2) snus; and 3) dissolvable tobacco products. Chewing tobacco, snuff, or dip users were respondents who reported using the product at least 20 times during their lifetime and now used it “every day” or “some days.” Snus or dissolvable tobacco product users were respondents who reported using each respective product at least once during their lifetime and now used it “every day” or “some days.”

††† *Northeast*: Connecticut, Maine, Massachusetts, New Hampshire, New Jersey, New York, Pennsylvania, Rhode Island, and Vermont. *Midwest*: Illinois, Indiana, Iowa, Kansas, Michigan, Minnesota, Missouri, Nebraska, North Dakota, Ohio, South Dakota, and Wisconsin. *South*: Alabama, Arkansas, Delaware, District of Columbia, Florida, Georgia, Kentucky, Louisiana, Maryland, Mississippi, North Carolina, Oklahoma, South Carolina, Tennessee, Texas, Virginia, and West Virginia. *West*: Alaska, Arizona, California, Colorado, Hawaii, Idaho, Montana, Nevada, New Mexico, Oregon, Utah, Washington, and Wyoming.

**TABLE 2:** Percentage of persons aged ≥18 years who were “every day,” “someday,” or “rarely” tobacco users among those who met established thresholds, by tobacco product and selected characteristics — National Adult Tobacco Survey, United States, 2012–2013

	Any tobacco product*		Any combustible tobacco product†		Cigars/Cigarillos/Filtered little cigars§		Regular pipe**		Water pipe/Hookah††		Electronic cigarettes§§		Smokeless tobacco¶¶	
							
Characteristics	%	(95% CI)	%	(95% CI)	%	(95% CI)	%	(95% CI)	%	(95% CI)	%	(95% CI)	%	(95% CI)
**Overall**	**25.2**	**(24.7–25.7)**	**22.9**	**(22.4–23.4)**	**5.8**	**(5.5–6.1)**	**0.9**	**(0.8–1.0)**	**3.9**	**(3.6–4.1)**	**4.2**	**(3.9–4.5)**	**3.8**	**(3.6–4.0)**
**Sex**														
Men	31.8	(31.0–32.6)	27.9	(27.1–28.8)	10.1	(9.5–10.7)	1.6	(1.4–1.8)	4.8	(4.4–5.2)	4.7	(4.3–5.1)	7.1	(6.6–7.5)
Women	17.5	(16.9–18.2)	16.8	(16.2–17.4)	1.5	(1.3–1.7)	0.2	(0.1–0.2)	2.7	(2.4–3.1)	3.6	(3.3–3.9)	0.4	(0.3–0.5)
**Age group (years)**														
18-24	35.2	(33.3–37.1)	32.2	(30.3–34.1)	8.9	(7.8–10.1)	1.2	(0.8–1.5)	18.2	(16.7–19.7)	8.3	(7.2–9.4)	6.6	(5.7–7.5)
25-44	29.5	(28.5–30.5)	27.0	(26.0–28.0)	7.4	(6.8–8.0)	1.0	(0.8–1.3)	3.9	(3.5–4.3)	5.0	(4.5–5.5)	5.1	(4.6–5.6)
45-64	24.5	(23.7–25.3)	22.1	(21.4–22.9)	4.9	(4.5–5.3)	0.7	(0.6–0.9)	0.4	(0.3–0.5)	3.4	(3.1–3.8)	2.7	(2.4–3.0)
≥65	10.6	(10.0–11.2)	9.4	(8.8–10.0)	2.0	(1.7–2.3)	0.6	(0.5–0.7)	—¶		1.1	(0.9–1.3)	1.2	(1.0–1.4)
**Race/Ethnicity**														
White, Non-Hispanic	24.6	(23.9–25.2)	21.8	(21.2–22.4)	5.6	(5.2–5.9)	0.9	(0.7–1.0)	3.6	(3.3–3.9)	4.4	(4.1–4.8)	4.4	(4.0–4.7)
Black, Non-Hispanic	25.5	(23.6–27.3)	24.4	(22.5–26.2)	6.5	(5.3–7.6)	—¶		2.0	(1.4–2.7)	1.8	(1.2–2.4)	1.3	(0.9–1.8)
Asian, Non-Hispanic	12.5	(9.7–15.3)	12.3	(9.4–15.1)	2.1	(0.9–3.3)	—¶		5.0	(3.1–7.0)	1.8	(0.7–2.9)	0.2	(0.1–0.4)
Other, Non-Hispanic	36.7	(34.4–39.0)	33.3	(31.0–35.5)	9.6	(8.1–11.0)	2.7	(1.9–3.5)	5.6	(4.4–6.8)	7.4	(6.2–8.7)	6.3	(5.1–7.5)
Hispanic	20.2	(18.7–21.7)	19.3	(17.8–20.8)	4.4	(3.6–5.2)	0.4	(0.2–0.7)	4.6	(3.8–5.4)	3.3	(2.6–4.0)	1.7	(1.2–2.1)
**U.S. Census region** ***														
Northeast	23.7	(22.4–25.1)	21.6	(20.3–22.9)	5.8	(5.0–6.6)	1.1	(0.7–1.4)	3.9	(3.1–4.6)	3.9	(3.2–4.6)	3.1	(2.5–3.7)
Midwest	27.7	(26.4–28.9)	24.6	(23.5–25.8)	6.4	(5.7–7.1)	1.2	(0.9–1.5)	4.0	(3.4–4.6)	4.7	(4.1–5.3)	5.4	(4.8–6.1)
South	26.4	(25.5–27.4)	24.0	(23.1–24.9)	6.2	(5.7–6.7)	0.9	(0.7–1.1)	3.5	(3.0–3.9)	4.5	(4.0–4.9)	4.2	(3.7–4.6)
West	23.3	(22.4–24.1)	21.4	(20.5–22.3)	5.1	(4.6–5.6)	0.7	(0.5–0.9)	4.3	(3.8–4.8)	3.9	(3.5–4.3)	2.9	(2.6–3.2)
**Education**														
0–12 years (no diploma)	30.1	(28.2–32.0)	28.0	(26.1–29.8)	5.9	(4.9–6.9)	1.2	(0.8–1.6)	2.1	(1.4–2.9)	4.0	(3.1–4.8)	3.6	(2.9–4.3)
GED	47.3	(43.0–51.6)	45.0	(40.7–49.4)	10.1	(7.0–13.2)	—¶		3.9	(2.0–5.8)	8.1	(5.5–10.7)	6.7	(4.5–8.9)
High School diploma	27.8	(26.6–28.9)	24.8	(23.7–25.9)	6.2	(5.6–6.9)	0.7	(0.5–0.9)	4.0	(3.4–4.6)	5.0	(4.5–5.6)	4.9	(4.4–5.5)
Some college, no diploma	28.5	(27.2–29.8)	26.5	(25.2–27.7)	6.8	(6.0–7.6)	1.1	(0.8–1.4)	6.4	(5.6–7.2)	5.4	(4.7–6.1)	3.8	(3.2–4.4)
Associate degree	24.9	(23.6–26.2)	22.3	(21.0–23.6)	5.5	(4.8–6.2)	0.8	(0.6–1.1)	3.1	(2.5–3.7)	5.1	(4.4–5.8)	3.7	(3.1–4.3)
Undergraduate degree	16.0	(15.2–16.9)	14.1	(13.3–14.9)	4.5	(4.0–5.0)	0.7	(0.5–0.9)	3.7	(3.2–4.2)	2.2	(1.8–2.5)	2.7	(2.3–3.1)
Graduate degree	10.2	(9.4–11.0)	9.2	(8.5–10.0)	3.4	(2.9–3.9)	0.5	(0.3–0.7)	2.0	(1.5–2.4)	0.9	(0.7–1.2)	1.1	(0.8–1.4)
**Annual household income ($)**														
<20,000	32.7	(30.9–34.5)	30.4	(28.6–32.2)	7.4	(6.4–8.5)	1.4	(1.0–1.8)	3.4	(2.6–4.1)	5.4	(4.5–6.3)	3.2	(2.6–3.9)
20,000–49,999	28.5	(27.5–29.6)	26.4	(25.3–27.4)	5.7	(5.1–6.2)	0.7	(0.6–0.9)	3.6	(3.1–4.1)	4.6	(4.1–5.1)	3.8	(3.4–4.3)
50,000–99,999	23.5	(22.5–24.5)	20.8	(19.9–21.8)	5.4	(4.8–6.0)	1.0	(0.7–1.3)	3.9	(3.3–4.4)	4.5	(3.9–5)	4.3	(3.8–4.8)
≥100,000	18.2	(17.1–19.3)	15.8	(14.7–16.8)	6.3	(5.6–7.1)	0.6	(0.4–0.8)	3.9	(3.3–4.5)	3.1	(2.6–3.6)	3.7	(3.2–4.3)
Unspecified	24.8	(23.6–26.0)	23.0	(21.8–24.2)	5.1	(4.5–5.7)	0.9	(0.6–1.1)	4.6	(4.0–5.3)	3.8	(3.2–4.3)	3.4	(2.8–3.9)
**Sexual orientation**														
Heterosexual/straight	24.4	(23.8–25.0)	22.1	(21.5–22.6)	5.7	(5.4–6.0)	0.8	(0.7–0.9)	3.5	(3.2–3.8)	4.1	(3.8–4.3)	3.8	(3.5–4.0)
LGBT	35.8	(32.6–39.0)	34.3	(31.1–37.5)	7.3	(5.4–9.1)	—¶		10.7	(8.5–12.9)	9.7	(7.6–11.8)	2.7	(1.6–3.9)
Unspecified	28.0	(26.3–29.7)	25.6	(23.9–27.3)	6.2	(5.2–7.1)	1.4	(1.0–1.8)	4.8	(3.9–5.6)	3.7	(3.0–4.5)	4.1	(3.3–4.9)

**Abbreviations:** CI = confidence interval; GED = General Education Development certificate; LGBT = lesbian, gay, bisexual, or transgender.

* Any tobacco use was defined as “every day” or “some days” use of cigarettes; and/or “every day,” “some days,” or “rarely” use of cigars, cigarillos, or filtered little cigars; pipes; water-pipes/hookahs; electronic cigarettes; smokeless tobacco (snus, dissolvable tobacco products, or snuff, chewing tobacco or dip). Cigarettes not presented separately because the questionnaire only assessed “every day” or “some days” cigarette smoking. Cigarette users included in the “any tobacco product” measure includes those who reported smoking at least 100 cigarettes during their lifetime and now smoked “every day” or “some days.”

† Any combustible tobacco use was defined as “every day” or “some days” use of cigarettes; and/or “every day,” “some days,” or “rarely” use of cigars, cigarillos, or filtered little cigars; pipes; or water pipes/hookahs. Cigarette users included in the “any combustible tobacco product” measure include those who reported smoking at least 100 cigarettes during their lifetime and now smoked “every day” or “some days.”

§ Reported smoking at least 50 cigars, cigarillos, or filtered little cigars during their lifetime and now smoked “every day” or “some days” or “rarely.”

¶ Estimate not presented because relative standard error ≥30%.

** Reported smoking a regular pipe filled with tobacco at least 50 times during their lifetime and now smoked “every day” or “some days” or “rarely.”

†† Reported smoking tobacco in a hookah at least once during their lifetime and now smoked “every day” or “some days” or “rarely.”

§§ Reported smoking electronic cigarettes at least once during their lifetime and now smoked “every day” or “some days” or “rarely.”

¶¶ Smokeless tobacco users were defined using three product types: 1) chewing tobacco, snuff, or dip; 2) snus; and 3) dissolvable tobacco products. Chewing tobacco, snuff, or dip users were respondents who reported using the product at least 20 times during their lifetime and now used it “every day,” “some days,” or “rarely.” Snus or dissolvable tobacco product users were respondents who reported using each respective product at least once during their lifetime and now used it “every day” or “some days” or “rarely.”

*** *Northeast:* Connecticut, Maine, Massachusetts, New Hampshire, New Jersey, New York, Pennsylvania, Rhode Island, and Vermont. *Midwest:* Illinois, Indiana, Iowa, Kansas, Michigan, Minnesota, Missouri, Nebraska, North Dakota, Ohio, South Dakota, and Wisconsin. *South:* Alabama, Arkansas, Delaware, District of Columbia, Florida, Georgia, Kentucky, Louisiana, Maryland, Mississippi, North Carolina, Oklahoma, South Carolina, Tennessee, Texas, Virginia, and West Virginia. *West:* Alaska, Arizona, California, Colorado, Hawaii, Idaho, Montana, Nevada, New Mexico, Oregon, Utah, Washington, and Wyoming.

## Footnotes

* Participants with missing responses for any of the assessed tobacco products (1.6% of respondents) were excluded.

† *Northeast*: Connecticut, Maine, Massachusetts, New Hampshire, New Jersey, New York, Pennsylvania, Rhode Island, and Vermont. *Midwest*: Illinois, Indiana, Iowa, Kansas, Michigan, Minnesota, Missouri, Nebraska, North Dakota, Ohio, South Dakota, and Wisconsin. *South*: Alabama, Arkansas, Delaware, District of Columbia, Florida, Georgia, Kentucky, Louisiana, Maryland, Mississippi, North Carolina, Oklahoma, South Carolina, Tennessee, Texas, Virginia, and West Virginia. *West*: Alaska, Arizona, California, Colorado, Hawaii, Idaho, Montana, Nevada, New Mexico, Oregon, Utah, Washington, and Wyoming.

§ Additional information available at http://www.fda.gov/tobaccoproducts/guidancecomplianceregulatoryinformation/ucm246129.htm.

¶ Additional information available at http://www.fda.gov/aboutfda/reportsmanualsforms/reports/economicanalyses/ucm394922.htm.

** Additional information available at http://www.tobaccofreekids.org/what_we_do/state_local/tobacco_settlement.
