# Errata

**Published:** 2014-07-04

## Vol. 63, No. 25

In the report, “Tobacco Product Use Among Adults — United States, 2012–2013,” one of the sexual orientation categories was incorrectly listed as lesbian, gay, bisexual, or transgender (LGBT). For the 2012–2013 National Adult Tobacco Survey, respondents could self-identify as lesbian, gay, or bisexual (LGB); the measure did not specifically assess whether a respondent was transgender. In the tables, on pages 543 and 544, the second listing under “Sexual orientation” should be **LGB**, defined as **LGB = lesbian, gay, or bisexual**. On page 545, the final sentence should read, “By sexual orientation, prevalence was higher among **lesbian, gay, or bisexual (LGB)** adults (30.8%) than heterosexual/straight adults (20.5).” On page 546, the second sentence under “Discussion” should read, “Any tobacco use was greater among men, younger adults, non-Hispanic other adults, those living in the Midwest and South, those with less education and income, and **LGB** adults.”

## Vol. 63, No. SS–4

In the MMWR Surveillance Summary “Youth Risk Behavior Surveillance — United States, 2013,” the title for Table 23 on page 72 was incorrect. It should read, “TABLE 23. Percentage of high school students **who felt sad or hopeless**,^*,†^ by sex, race/ethnicity, and grade — United States, Youth Risk Behavior Survey, 2013.”

